# Breast cancer in the post-genomic era

**DOI:** 10.1038/sj.bjc.6605681

**Published:** 2010-06-29

**Authors:** C Swanton

**Affiliations:** 1 CR-UK Translational Cancer Therapeutics Laboratory, London Research Institute, London, UK; 2 Royal Marsden Hospital, Breast and Drug Development Units, Sutton, UK

Few tumour types have received as much molecular analysis as breast cancer over the past 10 years. Impressive progress has been made in the analysis and interpretation of genomic data sets, the identification of novel mechanisms of breast cancer susceptibility, growth and development and the parallel integration of these molecular data to test novel therapeutic approaches for patients with this disease within clinical trials. This book, edited by Giordano and Normanno with individual chapters from experts in the field, successfully integrates this diverse field of progress within one volume, suitable for both medical professionals with an interest in breast cancer management and scientists with an interest in translational oncology. The book covers an impressive field, guiding the reader through scientific developments in breast tumour biology, cell-cycle control and growth factor signalling, to the complexities of stromal/tumour interactions, mammary gland development and stem cell biology, through to the clinical implications of basic research.

A consistent feature of the book is the elegant way in which the reader is led from the basic science observations underpinning the evidence through to the clinical implications of bench-side research. Several chapters review developments in the field from a clinical practice perspective. Chapter 3 explores the implications of developments in breast cancer genetics and its application in clinical practice, succinctly explained by Russo and colleagues. The authors cover a broad field, describing the molecular portrait of breast cancer using microarray analysis (also reviewed in detail in chapter 8) of gene and microRNA expression and the difficulties implementing such techniques into clinical practice, recently emphasised by Weigelt *et al* (2010). The authors present a useful section on oncogenetic counselling and genotype/phenotype relationship discussing the implications of distinct BRCA1/2 sequence variants and disease association.

Modelling breast cancer using transgenic mice is discussed in detail in chapter 7 by Dillon and Muller. The authors present structured and balanced examples supporting the use of animal models while expressing caution concerning the key differences between mouse and human mammary stromal tissue, patterns of cancer spread and the predominant oestrogen receptor negativity of most animal models. A future vision of developments in this field is presented, through the modelling of stromal/tumour interactions and the design of animal models that more robustly reflect hormone receptor profiles and patterns of spread witnessed in human breast cancer.

The final four chapters of the book discuss novel targets in breast cancer, their therapeutic implications and clinical trial considerations when developing drugs targeting these molecules. Lee and colleagues discuss the complexities of TGF-*β* signalling and the Jekyll and Hyde character of this signalling cascade in breast cancer mediating both tumour suppressor and oncogenic pathway activation and how this may impact on the successful targeting of this network. Szyf reviews epigenetic modification through the modification of gene expression through regional DNA hypermethylation in chapter 10. DNA methylation is associated with the silencing of tumour suppressor gene expression and widespread hypomethylation patterns involving metastasis-associated genes are commonly witnessed in solid tumours. This chapter presents a balanced view associated with therapeutic strategies aimed at modulating methyltransferase activity in breast cancer, detailing the complexity of this process mediated by the DNA methyltransferase family and their targeting to specific DNA positions through interaction with sequence recognition factors. The author discusses practical considerations when attempting to influence the activity of this process, as although DNA methyltransferase blockade may activate tumour suppressor gene expression, the paradoxical activation of metastasis-associated gene expression could also be unleashed.

Chapter 11, ‘Signal transduction inhibitors in the treatment of breast cancer’ by Maiello and colleagues, discusses the clinical trial results associated with the specific targeting of growth factor receptor pathways in breast cancer. Despite the excitement of the introduction of such strategies into clinical trial development in breast cancer, the authors emphasise that clinical activity associated with notable monotherapy strategies has been disappointing. Redundancy of growth factor signalling pathways and inter-patient tumoural heterogeneity are likely to contribute to these disappointing results.

The only potential criticism of such a book is the rate at which this field is progressing and how rapidly a second edition is required to keep up with developments. Perhaps the second edition will consider the impact of next-generation sequencing technologies on our understanding of the complexity of breast cancer tumourigenesis and tumour heterogeneity and the post-genomic functional annotation of the breast cancer genome using RNA interference strategies. In summary, *Breast Cancer in the Post-Genomic Era* will be highly relevant to both scientists and oncology health professionals requiring a practical overview of the field of translational breast cancer medicine.
